# Effect of Xanthan, Guar, and Carrageenan Gums on the Physicochemical Properties of Hypoallergenic Pea Protein-Based Dysphagia-Friendly Matrices

**DOI:** 10.3390/foods15020284

**Published:** 2026-01-13

**Authors:** Huaiwen Yang, Chi-Chung Hua, Po-Hsun Huang

**Affiliations:** 1 Department of Food Science, National Chiayi University, Chiayi City 60004, Taiwan; s1130422@mail.ncyu.edu.tw; 2 Department of Chemical Engineering, National Chung Cheng University, Chiayi County 621301, Taiwan; chmcch@ccu.edu.tw

**Keywords:** dysphagia, xanthan gum, guar gum, carrageenan, rheological properties, texture analysis

## Abstract

Due to the allergenicity of soy protein, this study aimed to develop a hypoallergenic, dysphagia-friendly matrix using pea protein isolate. We investigated the effects of three hydrocolloid thickeners—xanthan gum (XG), guar gum (G), and carrageenan (C)—at various concentrations on the matrices’ rheological properties, textural characteristics, and dysphagia diet classification. The unthickened pea protein base was unstable, exhibiting rapid phase separation and low viscosity, unsuitable for dysphagia diets. The addition of XG (0.4–0.6 g), G (0.5–1.0 g), and C (0.8–1.2 g) successfully produced food matrices meeting the slightly, mildly, and moderately thick levels of the Japanese Society of Dysphagia Rehabilitation (JSDR) framework. However, discrepancies were noted between instrumental viscosity and syringe flow test classifications. Rheological analysis revealed that XG samples were in elastic (*G*′ > *G*″) domain in the linear viscoelastic region (LVR) and exhibited shear-thinning behavior. In contrast, G and C samples were in viscous (*G*″ > *G*′) domain. Frequency sweeps characterize XG samples as weak gels, G samples as dilute polymer solutions, and C samples as gel-like structures. Texture profile analysis further showed that xanthan gum imparted the highest firmness and thickness, whereas guar gum provided the best flowability.

## 1. Introduction

Advances in medical technology have increased human lifespans [1]. However, older adults face greater risks of various health issues due to factors like immune system aging and the rising incidence of chronic conditions [1]. Proper nutrition is essential for managing these health problems. A common issue among this group is oropharyngeal dysphagia [2], which makes swallowing food and liquids difficult or painful. This condition often results from physiological issues such as esophageal strictures or neuromuscular disorders [2], which can cause inadequate nutrient intake and raise the risk of disease. Age-related degeneration of laryngeal and esophageal muscles further heightens the likelihood of swallowing difficulties [3]. Therefore, using food thickeners to modify textures for those with dysphagia is widely regarded as an effective intervention [4].

Rheology provides parameters to assess and understand the characteristics of foods suitable for people with dysphagia [5]. This improves their taste and safety [5]. Rheology is used to measure and analyze factors like viscosity and viscoelasticity, which are crucial for how food behaves in the mouth and throat during swallowing [6]. Thickeners modify the flow properties of foods and liquids [7], making them easier and safer for individuals with dysphagia to swallow [5]. In diets for the elderly, thickeners are often employed to alter food texture and prevent dysphagia-related issues [7]. According to earlier studies, samples thickened with xanthan gum (XG) display weak-gel-like behavior [8], while those thickened with guar gum (G) behave like dilute polymer solutions [9]. Samples thickened with carrageenan (C) exhibit gel-like properties [10,11].

Dysphagia can negatively impact a person’s health and quality of life [12]. In severe cases, individuals may be unable to eat normally [12]. This can lead to weight loss, malnutrition, airway blockage, and pneumonia [12]. Malnutrition often results from insufficient protein intake [12], as proteins are vital parts of tissues and cells [13]. They play roles in many metabolic and physiological processes [13]. Additionally, in some populations, certain proteins can trigger allergic reactions [14]. A protein allergy is an immune response that wrongly identifies specific proteins as harmful [14]. This can cause symptoms such as itchy skin, redness, swelling, vomiting, and low blood pressure [13]. These symptoms may make it difficult for affected populations to meet their daily protein needs [14]. As a result, alternative protein sources are necessary. Pea protein isolate (PPI) is a complete protein high in essential amino acids [14]. Furthermore, pea protein is free from gluten, lactose, cholesterol, and common food allergens [14], making it highly suitable for vegetarians and people with protein allergies [14].

To standardize the consistency of these thickened matrices, the Japanese Society of Dysphagia Rehabilitation (JSDR) 2013 established a classification system for thickened liquids. This system categorizes fluids into three primary levels: JSDR 1 (mildly thick), JSDR 2 (moderately thick), and JSDR 3 (highly thick) [15]. Each level corresponds to specific viscosity ranges and flow behaviors, allowing clinicians to match food consistency with the specific physiological needs and swallowing safety of the patient. Although earlier research has documented the distinct thickening behaviors of xanthan, guar, and carrageenan gums in various solutions [10,15,16], a significant gap remains in the literature regarding their use within plant-based protein systems. Specifically, studies focusing on a pea protein isolate (PPI)-based formulation are especially limited. The general properties of these gums are well understood; their behavior within a pea protein-based dysphagia matrix remains an area of active research with significant structural and safety implications.

To address this gap, this study aims to develop new, hypoallergenic protein matrices suitable for individuals with dysphagia. The main objectives are to create formulations using different ratios of these three common hydrocolloids with pea protein. Then, the study will analyze the rheological behavior and textural properties of these formulations. A key part of this work is to explore the relationship between these properties and the levels set by the International Dysphagia Diet Standardisation Initiative (IDDSI) framework. This study uses recent findings on rheological thermal resistance [15] and the structure of carrageenan gels [10] to provide essential data for the development of safe nutritional products.

## 2. Materials and Methods

### 2.1. Preparations of the Sample Matrices

The hydrocolloid concentrations were chosen to achieve three distinct consistency levels (L1, L2, and L3) defined by the Japanese Society of Dysphagia Rehabilitation (JSDR) 2013. These levels represent standardized viscosity ranges used in clinics to ensure swallowing safety in dysphagia patients.

Three low-allergenic pea protein formulations were utilized to achieve the thin (JSDR 1), medium (JSDR 2), and thick (JSDR 3) consistency levels as defined by the Japanese Society of Dysphagia Rehabilitation [15]. Pea protein isolate (PPI) was sourced from Yantai Tuell Biotech Co., Ltd. (Liaocheng, China). According to the manufacturer’s specifications, the PPI contained 85.6% protein (dry basis) and 6.43% moisture, with a pH of 7.2.

Each formulation was based on 15 g of pea protein isolate mixed with specific amounts of xanthan gum (XG), guar gum (G), and carrageenan (C). The JSDR 1 formulation contained 0.4 g of XG, 0.5 g of G, and 0.8 g of C; the JSDR 2 formulation contained 0.5 g of XG, 0.8 g of G, and 1.0 g of C; and the JSDR 3 formulation contained 0.6 g of XG, 1.0 g of G, and 1.2 g of C as listed in Table 1. For each preparation, the dry powder mixture was incorporated with reverse osmosis water to a final weight of 200 g. Then it was homogenized at 6000 rpm for 3 min at room temperature (T 25 digital, IKA-Werke GmbH & Co. KG, Staufen, Germany).

Three low-allergenic pea protein formulations were used to achieve the thin (JSDR 1), medium (JSDR 2), and thick (JSDR 3) consistency levels, as defined by the Japanese Society of Dysphagia Rehabilitation [17]. The specific concentrations of xanthan gum (0.4–0.6 g), guar gum (0.5–1.0 g), and carrageenan (0.8–1.2 g) were chosen based on preliminary trials to determine the effective range needed to stabilize the 15 g pea protein isolate base while meeting these distinct texture levels. The JSDR framework was selected for its accurate instrumental viscosity categories and its clinical utility as measured by the syringe flow test. Additionally, the IDDSI framework was used to provide a global context for the results, ensuring that the developed hypoallergenic matrices conform to internationally recognized safety and consistency standards for dysphagia management.

The specific concentrations of guar gum (0.5, 0.8, and 1.0 g) were chosen based on preliminary tests to ensure the formulations fit within the viscosity ranges of JSDR Levels 1, 2, and 3, respectively. Mudgil et al. [8] reported that guar gum is a galactomannan that mainly thickens through molecular entanglement. During our initial formulation tests, a concentration of 0.5 g was enough to reach the maximum viscosity of the JSDR 1 category for this high-protein matrix. The use of g for the JSDR 1 level shows the strong thickening ability of guar gum in the presence of pea protein, where higher concentrations would make the matrix too thick for the intended level.

### 2.2. Sedimentation Index (SI)

The sedimentation index (SI) was assessed using a modified protocol based on the report by Yang et al. [16]. Briefly, place each sample into a 50 mL centrifuge tube and maintain it at room temperature without disturbance. The sedimentation index test was performed every 5 min to assess the progression of sedimentation over time. The SI was calculated according to Equation (1):

(1) SI (%) = (V_sediment_/V_total_) × 100

where V_sediment_ represents the volume of the sediment layer, and V_total_ is the initial total volume of the sample.

### 2.3. Dysphagia-Friendly Evaluations

#### 2.3.1. Rheological Measurement

Rheological properties were analyzed with a TA Instruments DHR-2 stress-controlled rheometer (double-gap cylinder geometry) at 25 °C. A strain sweep (0.01–100% strain at 1 Hz) identified the linear viscoelastic region, after which a frequency sweep (0.1–100 Hz at 1% strain) measured the storage (G′) and loss (G″) moduli. Steady-state viscosity was determined by applying a constant shear rate of 50 s^−1^ [17], with average values used for sample classification in accordance with JSDR standards [17].

#### 2.3.2. Empirical Syringe Flow Test

**Figure A1:**
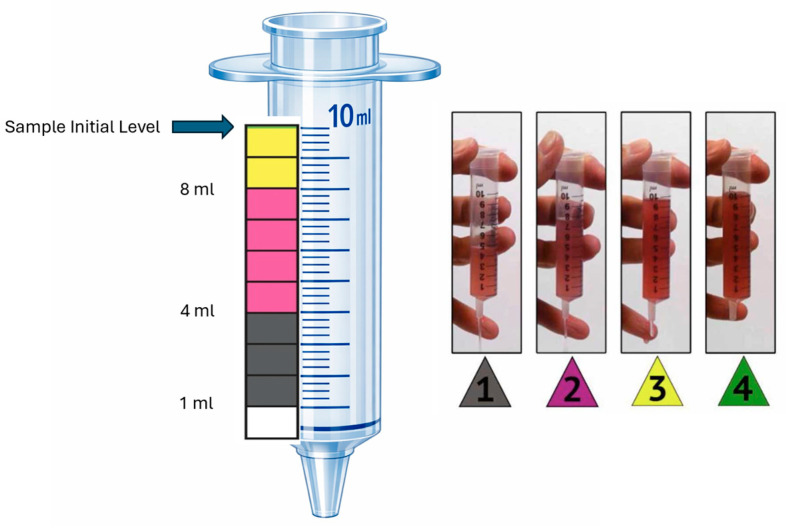
Visual depiction of the IDDSI flow test for pea protein isolate (PPI) matrices. The test used a 10 mL syringe with a 0–10 mL scale, measuring 61.5 mm in length. The remaining volume after 10 s of flow indicates the consistency level: Level 1 (Thin): 1–4 mL remaining; Level 2 (Mildly Thick): 4–8 mL remaining; Level 3 (Moderately Thick): 8–10 mL remaining; Level 4 (Extremely Thick): 10 mL remaining (no flow).

Each sample’s flow properties were evaluated with the syringe flow test according to the guidelines set out by the JSDR [15]. The empirical flow behavior was tested using the IDDSI flow test with a 10 mL syringe (0–10 mL scale length = 61.5 mm). Results were recorded as the volume of sample remaining in the syringe after 10 s of flow (Appendix A Figure A1).

For this procedure, a 10 mL Luer-slip tip syringe with the plunger removed served as the apparatus. To fill the syringe to the 10 mL mark, the outlet was initially blocked. After filling, the outlet was opened to allow the sample to flow downward by gravity for precisely 10 s. After this step, the outlet was blocked again, and the volume of sample remaining in the syringe (in milliliters) was determined. This measurement helped determine the sample’s consistency based on standards set by both the JSDR and the International Dysphagia Diet Standardisation Initiative (IDDSI) framework [18].

### 2.4. Texture Profile Analysis (TPA)

A TA.XTplus texture analyzer (Stable Micro Systems, Godalming, Surrey, UK) was used to evaluate the texture of the samples, following a modified method inspired by [19]. A 40 mm diameter disc probe was used to perform a back extrusion test (A/BE). Exponent Lite software (version 6) was used to collect and analyze data, assessing seven textural properties: liquidity, firmness, uniformity, consistency, cohesiveness, viscosity index, and subsidiary.

### 2.5. Statistical Analysis

Experiments were repeated three times, and results are presented as the mean ± SD. Data were analyzed using SPSS 19.0; one-way ANOVA was used to evaluate group differences, and Duncan’s test was employed for post hoc comparisons (*p* < 0.05). SigmaPlot^®^ (version 10, SYSTAT Software, Inc., Palo Alto, CA) was employed to present the figure results.

## 3. Results and Discussion

### 3.1. Incorporation of Thickeners in Low-Allergenic Pea Protein Isolate (PP) Matrix

#### 3.1.1. Sedimentation Index of Matrices Without Thickening

**Figure A2:**
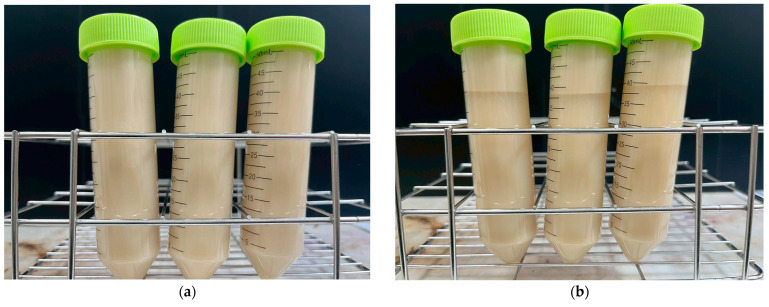
Sedimentation results of the native PPI at 0 (**a**) and 10 (**b**) min.

Before adding thickening agents, it was necessary to assess the baseline physical stability of the standalone low-allergenic pea protein isolate (PP) matrix. The native pea protein matrix showed poor stability, with quick and noticeable sedimentation (results at 0 and 10 min shown in Appendix A Figure A2). As shown in Table 2, phase separation into a clear liquid supernatant and a protein-rich sediment occurred within the first 5 min. Sedimentation was complete within 30 min, and the sediment volume remained stable thereafter. This behavior is typical of plant-based protein dispersions, which often have limited solubility and poor suspension stability in water [20]. For people with dysphagia, who usually take longer to eat, this phase separation is a safety issue, as the inconsistent textures of the separated layers could raise the risk of choking or aspiration.

#### 3.1.2. Sedimentation Stability of Thickened Low-Allergenic PP Matrices

The thickening with various gums at different concentrations (Table 1) effectively stabilized the low-allergenic pea protein isolate (PPI) matrices designed for individuals with dysphagia (Table 2); these results align with the IDDSI syringe test results shown in Table 3. This physical stability is governed by distinct mechanisms depending on the hydrocolloid type. Sample thickened with X. Specifically, xanthan gum reported by Yang et al. [7] provides high yield stress and a weak-gel-like behavior even at low concentrations, which creates a network capable of effectively suspending insoluble protein particles against gravitational forces. Musampa et al. [9] reported that carrageenan contributes by demonstrating strong gel-like behavior, establishing a robust structural matrix that inhibits the phase separation typically encountered in plant-based protein systems. Furthermore, the synergy between these hydrocolloids and the pea protein isolate enhances the overall physical stability of the suspension. This is achieved by increasing the steric hindrance and electrostatic repulsion between particles, thereby maintaining a homogeneous consistency essential for the safe oral transit of dysphagia-friendly foods.”

Phase separation was absent in all samples within the first thirty minutes after thickening, which is essential for ensuring safe consumption by patients with swallowing difficulties. However, the thickened, low-allergenic PP matrices remained homogeneous throughout the 24 h (Table 4), demonstrating that our sample gums improve and maintain product stability (This consistent level of stability is crucial for dysphagia management, as phase separation—where liquid separates from the solid or thickener—raises the risk of aspiration (food or liquid entering the airway) during swallowing. Incorporating gum (thickener) ensures a uniform, safer texture for consumption).

### 3.2. JSDR and IDDSI Categorizations

According to literature, the human swallowing rate is approximately 50 s^−1^ [20]. As a result, food for various degrees of dysphagia is commonly classified based on its viscosity measured at this specific shear rate.

The JSDR measurement method is the same as the one described previously and is divided into thin consistency (50–150 cP), medium consistency (150–300 cP), and thick consistency (300–500 cP). From Table 4, it can be observed that different gums require different amounts to achieve the different viscosity levels of the JSDR classification. The usage amounts are in the order: Carrageenan > Guar Gum > Xanthan Gum. The data in the table indicates that minimal quantities of xanthan gum are sufficient to attain varying degrees of dysphagia suitability in beverages, which aligns with findings reported in prior studies [20,21]. The current cost per kilogram for the three thickening gums is: Xanthan Gum: 700 NTD, Guar Gum: 180 NTD, Carrageenan: 1200 NTD. Therefore, in product development, cost is also a consideration, alongside product characteristics.

The JSDR association added the syringe measurement method reported by Kayashita et al. [15]. This approach does not require advanced equipment, allowing medical staff, food preparers, and caregivers to classify thickened liquids by measuring the residual volume after a set time through a syringe. The classifications include thin consistency (2.2–7.0 mL), medium consistency (7.0–9.5 mL), and thick consistency (9.5–10.0 mL), enabling quick classification.

From Table 5, we observed that the classification of samples using the syringe measurement method slightly differs from that of the viscosity measurement method. Samples with the highest concentrations of Xanthan Gum, Guar Gum, and Carrageenan are considered thick because their viscosity measurements classify them as such. However, when using the syringe measurement method, the highest-concentration samples of these thickening gums are classified as medium consistency. Therefore, under the same standard system, different measurement methods can yield different results: XG0.6, G1.0, and C1.2 classified as category 2 in the syringe test instead of category 3 in the viscosity measurements. However, the matrices can still be safe to swallow. We recommend that the formula designers adjust the thickener concentrations to avoid such minor discrepancies.

The IDDSI thickened liquid classification is as follows: Level 0 Thin (0.0–1.0 mL), Level 1 Slightly Thick (1.0–4.0 mL), Level 2 Mildly Thick (4.0–8.0 mL), Level 3 Moderately Thick (8.0–10.0 mL), and Level 4 Extremely Thick (10 mL, does not flow out of the syringe at all). This is different from the JSDR classification of thin consistency (2.2–7.0 mL), medium consistency (7.0–9.5 mL), and thick consistency (9.5–10.0 mL). The corresponding IDDSI Level 1 Slightly Thick (1.0–4.0 mL) and Level 2 Mildly Thick (4.0–8.0 mL) are similar to the JSDR’s thin consistency (2.2–7.0 mL). However, IDDSI Level 3 Moderately Thick (8.0–10.0 mL) almost completely covers the JSDR’s medium consistency (7.0–9.5 mL) and thick consistency (9.5–10.0 mL). This results in different classifications in Table 6: only XG 0.4 in the xanthan gum group, and G0.8 and C1.0 have different classifications. Discrepancies were observed between instrumental viscosity (JSDR 3) and syringe flow test results (JSDR 2) for high-concentration samples (XG 0.6, G 1.0, and C 1.2) [10]. This has important practical implications: For Product Development, relying only on a fixed shear rate (50 s^−1^) may overestimate a matrix’s thickness in real-world scenarios [11]. Developers should formulate products to fall comfortably within the middle of a target range to account for these measurement variations [12]. For Caregivers and Clinical Staff, the syringe test offers a quick, equipment-free method to classify liquids at the bedside [13]. Since gravity-driven flow more accurately mimics the oral and pharyngeal stages of swallowing, the syringe result should be prioritized for immediate safety assessments when instrumental data conflicts [14]. Regarding the Safety Margins: Despite the discrepancies, all developed matrices remained homogeneous for 24 h, ensuring that the texture stays safe even if the specific classification level shifts slightly between testing methods [15].

### 3.3. Rheological Measurement

#### 3.3.1. XG-Thickened PP Matrices

**Figure 1:**
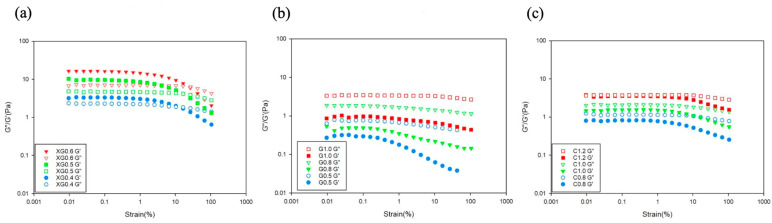
Strain-dependent rheological patterns (strain sweep: 0.01–100% at 1 Hz, 25 °C) of low-allergenic pea protein (15 g/100 g) matrices thickened with (**a**) xanthan gum, (**b**) guar gum, and (**c**) carrageenan across JSDR levels 1–3.

The strain-sweep results for thickened PP matrices with different xanthan gum (XG) concentrations are shown in Figure 1a. At all three concentrations, the linear viscoelastic region (LVER) of the XG-thickened pea protein beverages exhibited *G*′ *> G*″, indicating that the systems were in the elastic domain. As the strain increased, the sample’s behavior transitioned from *G*″ > *G*′ to *G*′ > *G*″, becoming a viscous domain and exhibiting shear-thinning behavior.

A “weak-strain overshoot” phenomenon was observed at higher strain values. This is attributed to the polymer chain structure resisting deformation under increasing strain, which leads to an increase in *G*″. As the sample experiences increased deformation, the polymer chains straighten and orient along the flow direction, which causes *G*″ to decrease [22]. As the xanthan gum concentration in the sample increased, the weak-strain overshoot became more pronounced, which is consistent with previous research (Yang et al., 2021) [7].

Figure 1a displays the strain sweep data for the PP matrices thickened with XG. For the XG 0.4 (JSDR1) group, the sample transitioned from critical gel characteristics to gel-like characteristics. However, at high frequencies, a crossover (*G*″ > *G*′) occurred, where viscous properties exceeded elastic properties, demonstrating liquid-like behavior. This is consistent with previous findings [8] and is a characteristic often observed in low-concentration xanthan gum samples. With increasing xanthan gum concentration, the XG 0.5 and XG 0.6 groups showed *G*′ > *G*″ with both *G*′ and *G*″ increasing with frequency, indicating weak-gel-like behavior.

#### 3.3.2. G-Thickened PP Matrices

**Figure 2:**
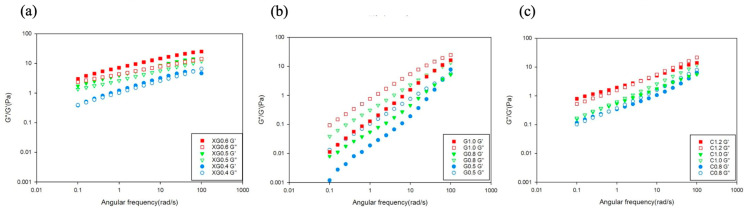
Frequency-dependent rheological patterns (frequency sweep from 0.1 to 100 rad/s at 1% strain, 25 °C) of low-allergenic pea protein (15 g/100 g) matrices thickened with (**a**) xanthan gum, (**b**) guar gum, and (**c**) carrageenan across JSDR levels 1–3.

The frequency-sweep results for pea protein matrices with different guar gum concentrations (G-thickened PP matrices) are shown in Figure 2b. At all three concentrations, the linear viscoelastic region of the G-thickened PP matrices showed *G*″ > *G*′, indicating that the systems were in the viscous domain. As both strain and guar gum concentration increased, the samples remained in the same viscous domain, consistent with previous findings [23].

The frequency sweep results for the G-thickened PP matrices are shown in Figure 2b. As the scanning frequency increased, the viscous modulus remained greater than the elastic modulus (*G*″ > *G*′), indicating that the emulsion solution was in a state of good fluidity, consistent with diluted polymer solutions. Guar gum (G) acts as a macromolecular biopolymer in aqueous solutions, and its frequency sweep results typically show the loss modulus (*G*″) being higher than the storage modulus (*G*′) [9].

#### 3.3.3. C-Thickened PP Matrices

The strain-sweep results for pea protein beverages with different carrageenan concentrations are shown in Figure 1c. At all three concentrations, the linear viscoelastic region of the carrageenan-thickened pea protein beverages showed *G*″ > *G*′, indicating that the systems were viscous-oriented. However, as the carrageenan concentration increased, the gap between *G*′ and *G*″ gradually narrowed, and in the C 1.2 (JSDR3) group, *G*′ and *G*″ are similar.

Figure 2c displays the frequency-sweep results for pea protein beverages thickened with carrageenan. For all groups, *G*′ > *G*″ at the initial (low) frequencies, but as the scanning frequency increased, a crossover occurred where the viscous modulus became greater than the elastic modulus (*G*″ > *G*′*)*. This demonstrates gel-like behavior and is consistent with previous research [10,11].

### 3.4. Texture Analysis

#### 3.4.1. Liquidity

Liquidity is defined as the gradient from zero to the yield point; a smaller value indicates better sample liquidity. As shown in Table 7, the liquidity values for the XG group were 6.24 g/s, 6.60 g/s, and 7.05 g/s, respectively. As the hydrocolloid concentration increased, the sample’s liquidity decreased. In the G group, a significant difference was observed between the JSDR 2 sample and the low- and high-concentration groups, with the medium-concentration sample exhibiting better liquidity than the other two. In the C group, increasing hydrocolloid concentration had no significant effect on sample liquidity; JSDR 1: G > XG > C, JSDR 2: G > XG = C, JSDR 3: Liquidity (best to worst): G > XG = C. (best to poorest).

#### 3.4.2. Firmness

Firmness is the maximum positive peak force on the curve, representing the sample’s firmness. As shown in Table 7, in the XG, G, and C groups, sample firmness increased with gum concentration, and there were significant differences among the concentration groups (*p* < 0.05). In the JSDR 1 group, the firmness from largest to smallest was XG > G = C. In the JSDR 2 group, it was XG > G = C. In the JSDR 3 group, it was XG = G > C.

The above results are consistent with previous literature, which shows that samples prepared with xanthan gum have higher firmness than those without xanthan gum [24].

#### 3.4.3. Uniformity

Homogeneity is the linear distance from the yield point to the maximum positive peak force, representing the smoothness of the sample. A smaller value indicates better internal homogeneity of the sample.

As shown in Table 7, there were no significant differences in homogeneity across all groups, indicating that the gum was evenly distributed in the samples during preparation and that the samples’ internal homogeneity was good.

#### 3.4.4. Consistency

Consistency is the positive peak area on the curve; a larger value indicates a thicker consistency. As shown in Table 7, in the XG, G, and C groups, sample consistency increased with gum concentration, and there were significant differences among the concentration groups (*p* < 0.05). In the JSDR 1 group, the consistency from largest to smallest was XG > G = C. In the JSDR 2 group, it was XG > G = C. In the JSDR 3 group, it was XG > G > C.

#### 3.4.5. Cohesiveness

Cohesiveness is the maximum negative peak force value on the curve, reflecting the strength of the intermolecular bonding within the sample. A larger value indicates greater cohesiveness. As shown in Table 7, sample cohesiveness increased with gum concentration in the XG, G, and C groups. This indicates that as the gum concentration in the sample increased, the internal intermolecular bonding became stronger. There were significant differences between the different concentration groups (*p* < 0.05). The cohesiveness from largest to smallest was: JSDR 1 group, XG > C > G; JSDR 2 group, XG > G > C; JSDR 3 group, G > XG > C.

#### 3.4.6. Viscosity

Viscosity is the area of the negative peak on the curve; a larger value indicates greater viscosity of the sample. As shown in Table 7, in the XG, G, and C groups, as the gum concentration increased, the sample viscosity also increased, and there were significant differences among the concentration groups (*p* < 0.05). In the JSDR 1 group, the consistency from largest to smallest was XG = C > G. In the JSDR 2 group, it was G > XG = C. In the JSDR 3 group, it was G > XG = C.

#### 3.4.7. Mouthcoating (Subsidiary)

Mouthfeel is the ratio of the maximum positive peak force to the maximum opposing peak force, indicating the ease with which the sample dissolves in the mouth. The results in Table 7 show no differences in the mouthfeel among the three XG concentration groups. In the G and C groups, as gum concentration increased, the sample’s mouthfeel also increased, and there were significant differences between the concentration groups (*p* < 0.05). In the JSDR 1 group, the mouthfeel difficulty, from most to least, was XG = C = G. In the JSDR 2 group, G > XG, while C showed no difference with either. In the JSDR 3 group, it was G > C > XG.

The systematic comparison of xanthan gum (XG), guar gum (G), and carrageenan (C) in a hypoallergenic pea protein (PP) matrix provides critical data for the development of safe, palatable, dysphagia-friendly matrices. The results clearly demonstrate that each hydrocolloid imparts a unique set of rheological and textural properties, which must be carefully matched to the target dysphagia classification. The strain-sweep analysis effectively characterized the fundamental viscoelastic nature of the thickened matrices. Xanthan Gum (XG): The LVR for all XG concentrations showed *G*′ > *G*″. This signifies in the elastic (solid-like) domain, consistent with XG’s ability to form a strong, ordered network, even in the presence of PP. The observation of shear-thinning behavior and the “weak-strain overshoot” phenomenon confirms that the XG structure resists initial deformation but rapidly breaks down under high strain, a beneficial property for easing the swallowing process. Guar Gum (G): In contrast, G samples remained in the viscous (*G*″ > *G*′) domain in the LVR. The frequency sweep further classified them as dilute polymer solutions, indicating that guar gum primarily acts as an effective non-gelling thickener, increasing the fluid’s resistance to flow but not significantly its elastic structure. Like G, C was viscous-oriented (G″ > G′) in the strain sweep LVR. However, the frequency sweep demonstrated gel-like behavior, where the elastic modulus (*G*′) dominated at low frequencies before a crossover occurred, indicating the formation of a recoverable, structured network. This is a crucial distinction from guar gum, as carrageenan provides a more structured matrix, especially at rest. Texture Profile Analysis (TPA) provided quantitative measurements corresponding to oral processing attributes. Xanthan Gum imparted the highest firmness and consistency across most JSDR categories. This suggests XG creates the most rigid and thickest feel, which is essential for maximizing bolus control and reducing pharyngeal flow speed, but may require more effort to swallow. Guar Gum provided the best liquidity (flowability). Crucially, G surpassed XG and C in both cohesiveness and viscosity in the medium (JSDR 2) and thick (JSDR 3) consistency groups This indicates that while G is more flowable at rest, its high concentration creates strong internal molecular bonding and resistance (viscosity) during extrusion, potentially optimizing bolus integrity and ease of movement. Guar Gum also resulted in the highest mouth-coating (Subsidiary) in the JSDR 2 and 3 groups. This attribute relates to how easily the sample dissolves in the mouth and its sensation. A higher mouth-coating may be desirable for certain dysphagia patients, as it provides a clearer sensory signal of the bolus and reduces the perception of a dry mouth, regarding formulation efficiency and classification discrepancies. The study highlighted practical implications regarding usage, cost, and classification: once in classification results between the instrumental viscosity measurement (rheometer at 50 s^−1^) and the empirical syringe flow test. Samples with the highest concentrations (XG0.6, G1.0, and C1.2) were rated “thick consistency” (JSDR 3) by viscosity measures, but “medium consistency” (JSDR 2) by the syringe test. This underscores the importance of using multiple assessment methods (rheology and empirical flow) in quality control to ensure products meet safety standards, as flow properties determined by gravity-driven tests may better mimic real-world swallowing mechanics than those from fixed-shear-rate viscosity measurements. As for concentration efficiency, the required usage amount to achieve target viscosities followed the order: Carrageenan > Guar Gum > Xanthan Gum. XG’s high thickening efficiency translates to lower material costs per batch, making it potentially the most cost-effective thickener. A key finding was the difference in classification results between the instrumental viscosity measurement (rheometer at 50 s^−1^) and the empirical syringe flow test. Specifically, the highest-concentration samples (XG0.6, G1.0, and C1.2) were classified as “thick consistency” (JSDR 3) by viscosity but only as “medium consistency” (JSDR 2) by the syringe test. This underscores the importance of using multiple assessment methods (rheology and empirical flow) in quality control to ensure products meet safety standards, as flow properties determined by gravity-driven tests may better mimic real-world swallowing mechanics than those from fixed-shear-rate viscosity measurements.

### 3.5. Discussion

Unlike soy protein isolate and dairy protein isolate (casein and whey), which are classified globally as major allergens and require strict labeling and facility segregation, yellow split peas (the source of PPI) are considered a “cleaner” and more “allergen-friendly” option [25]. Although rare legume sensitivities occur, PPI is widely recognized in food science as hypoallergenic and suitable for people with multiple dietary restrictions [26]. While the J SDR 2013 framework offers key clinical guidelines in Japan, the international dysphagia diet standardization initiative (IDDSI) manages global regions such as the Americas (United States and Canada), Europe (the United Kingdom, Ireland, and Germany), Oceania (Australia and New Zealand), Middle Eastern countries (United Arab Emirates and Saudi Arabia), and Asia (Singapore, Malaysia, and Hong Kong) to unify dysphagia management [17]. For clinical use, empirical syringe tests are preferred because of their simplicity. However, for developing new protein-based matrices, instrumental radiological assessment remains the scientific gold standard, as it provides accurate data on shear-thinning behavior essential for safe bolus transit.

The physical stability of pea protein isolate (PPI) matrices is essential for ensuring the safety of dysphagia-friendly products. In this study, the control PPI suspension exhibited significant instability, with the sedimentation index dropping to 38.6% within 30 min. Adding xanthan gum, guar gum, and carrageenan at the concentrations listed in Table 1 effectively stabilized the PPI matrices, consistent with previous findings that hydrocolloids enhance system stability by increasing water retention and forming soft, cohesive structures [27]. Specifically, xanthan gum promotes weak-gel-like behavior that effectively suspends protein particles [28]. Supramolecular studies further suggest that blending guar gum and xanthan gum at optimal ratios (e.g., 8:2 or 9:1) significantly increases zero-shear viscosity and yield stress, thereby improving stabilization compared with single-gum systems [27,28]. Therefore, future research could explore mixed gel systems.

The formulation strictly follows the 2013 standards established by the Japanese Society of Dysphagia Rehabilitation (JSDR). For example, using 0.05 g of guar gum (JSDR level 1) is necessary to prevent over-thickening caused by guar gum galactomannans, which mainly thicken through molecular entanglement [27]. Advanced rheological measurements reported in a recent study [28] show that pyruvate content in xanthan gum plays a key role in PPI–xanthan gum interactions, with lower pyruvate levels significantly reducing viscosity and mechanical strength [28]. Additionally, PPI-based matrices modified with about 0.5% (*w*/*w*) xanthan gum fall into IDDSI level 4 (pureed/extremely thick) classifications [27,29].

In our texture-modified diet demonstration, these recent insights provide a more detailed understanding of how gum chemistry influences the structural integrity of PPI-dense systems. Regional standards, such as those of the JSDR, were cross-validated against the IDDSI syringe test to assess global applicability [27]. However, recent literature indicates that for precise formulation design and 3D printing applications, critical rheological parameters—namely yield stress and storage modulus (G′)—remain the gold standard for predicting safe swallowing and bolus flow [28,30]. This dual-assessment approach ensures that low-allergenic PPI matrices meet the nutritional and safety needs of aging populations worldwide.

## 4. Conclusions

This study compared rheological and textural properties of formulations with xanthan gum (XG), guar gum, and carrageenan that meet dysphagia standards. Strain-sweep analysis showed that XG was predominantly elastic, whereas guar gum and carrageenan were more viscous. In frequency sweeps, XG at 0.4% behaved as a weak gel, shifting to liquid-like at high frequencies; guar gum acted as a dilute solution, and carrageenan remained gel-like. Texture profile analysis found XG had the greatest firmness and consistency, whereas guar gum provided superior flowability and mouth-coating (especially in JSDR 2 and 3). At low concentration (JSDR 1), XG led in cohesiveness and viscosity; at medium/high concentrations, guar gum surpassed XG in these metrics. Thus, hydrocolloid selection strongly affects viscoelastic traits and texture, which are important for safe swallowing.

## Figures and Tables

**Table 1:** Experimental sample formulation list *.

Formulation (Category)	Thickener + PP (g/100 g)
XG 0.4 (JSDR 1)	0.4 g XG + 15 g PP
XG 0.5 (JSDR 2)	0.5 g XG + 15 g PP
XG 0.6 (JSDR 3)	0.6 g XG + 15 g PP
G 0.5 (JSDR 1)	0.5 g G + 15 g PP
G 0.8 (JSDR 2)	0.8 g G +15 g PP
G 1.0 (JSDR 3)	1.0 g G + 15 g PP
C 0.8 (JSDR 1)	0.8 g C + 15 g PP
C 1.0 (JSDR 2)	1.0 g C + 15 g PP
C 1.2 (JSDR 3)	1.2 g C + 15 g PP

* XG: xanthan gum; G: guar bean gum; C: carrageenan; PP: pea protein isolate; data shown as mean ± SD (*n* = 3).

**Table 2:** Sedimentation changes of low-allergenic PP dysphagia-friendly matrices without thickening *.

Time (min)	Sedimentation Index (%)
5	90.0 ± 2.00
10	81.3 ±1.15
15	70.6 ± 1.15
20	60.0 ± 2.00
25	49.3 ± 1.15
30	38.6 ± 3.05

* Values are mean ± SD (*n* = 3); PP concentration is 15 g using reverse osmosis water as the aquatic base until the total weight reaches 200 g (7.5%), while 50 mL of each sample was measured.

**Table 3:** JSDR qualification and IDDSI categorizations of PP dysphagia-friendly matrices without thickening *.

Measuring Apparatus	Outcome	Level
Rheometer	7.79 ± 1.28 cP	JSDR N.Q.
10-mL syringe	Remaining volume	JSDR N.Q.
	0 mL	
	Remaining volume	IDDSI 0
	0 mL	

* Values are mean ± SD (*n* = 3); PP concentration is 15 g per 100 g reverse osmosis water; N.Q. stands for the sample not qualified as dysphagia-friendly.

**Table 4:** JSDR viscosity classification of low-allergenic PP dysphagia-friendly matrices employed with hydrocolloidal thickeners after 24 h *.

Sample Thickener	Viscosity (cP)	JSDR Level
XG0.4	141.5 ± 0.62	1
XG0.5	251.4 ± 0.95	2
XG0.6	307.0 ± 0.55	3
G0.5	91.7 ± 0.63	1
G0.8	187.4 ± 0.80	2
G1.0	367.3 ± 0.36	3
C0.8	139.1 ± 1.91	1
C1.0	216.8 ± 4.05	2
C1.2	366.4 ± 15.75	3

* XG: xanthan gum; G: guar bean gum; C: Carrageenan; values are mean ± SD (*n* = 3); the sample thickeners correspond to Table 1.

**Table 5:** JSDR Syringe residue classification of PP dysphagia-friendly matrices.

Sample Thickener	Remaining Volume (mL)	JSDR Category
XG0.4	6.8 ± 0.20	1
XG0.5	7.8 ± 0.10	2
XG0.6	8.8 ± 0.10	2
G0.5	5.4 ± 0.30	1
G0.8	8.6 ± 0.10	2
G1.0	9.4 ± 0.10	2
C0.8	7.2 ± 0.10	1
C1.0	8.6 ± 0.10	2
C1.2	9.4 ± 0.10	2

XG: xanthan gum; G: guar bean gum; C: Carrageenan; values are mean ± SD (*n* = 3); the sample thickeners correspond to Table 1.

**Table 6:** IDDSI Syringe residue classification of low-allergenic PP dysphagia-friendly matrices.

Sample Thickener	Remaining Volume (mL)	IDDSI Level
XG0.4	6.8 ± 0.20	2
XG0.5	7.8 ± 0.10	2
XG0.6	8.8 ± 0.10	3
G0.5	5.4 ± 0.30	2
G0.8	8.6 ± 0.10	3
G1.0	9.4 ± 0.10	3
C0.8	7.2 ± 0.10	2
C1.0	8.6 ± 0.10	3
C1.2	9.4 ± 0.10	3

XG: xanthan gum; G: guar bean gum; C: Carrageenan; values are mean ± SD (*n* = 3); the sample thickeners correspond to Table 1.

**Table 7:** Textural properties of thickened low-allergenic pea protein dysphagia-friendly matrices *.

sample		Liquidity (g/s)		
		JSDR1	JSDR2	JSDR3
XG		6.2 ± 0.09 ^B,b^	6.6 ± 0.28 ^AB,a^	7.0 ± 0.31 ^A,a^
G		6.8 ± 0.09 ^A,a^	6.2 ± 0.03 ^B,a^	6.9 ± 0.19 ^A,a^
C		5.7 ± 0.09 ^A,c^	5.8 ± 0.11 ^A,b^	5.9 ± 0.17 ^A,b^
sample		Uniformity (g.s)		
		JSDR1	JSDR2	JSDR3
XG		1.1 ± 0.74 ^A,a^	0.7 ± 0.45 ^A,a^	0.3 ± 0.32 ^A,b^
G		0.8 ± 0.69 ^A,a^	1.1 ± 0.67 ^A,a^	1.8 ± 0.56 ^A,a^
C		0.6 ± 0.58 ^A,a^	0.5 ± 0.41 ^A,a^	0.5 ± 0.27 ^A,b^
sample	Cohesiveness (g)			
	JSDR1		JSDR2	JSDR3
XG	−13.9 ± 0.11 ^A,c^		−15.6 ± 0.05 ^B,c^	−17.7 ± 0.32 ^C,b^
G	−11.6 ± 0.11 ^A,a^		−15.3 ± 0.05 ^B,b^	−20.5 ± 0.29 ^C,c^
C	−12.0 ± 0.11 ^A,b^		−13.9 ± 0.88 ^B,a^	−16.8 ± 0.43 ^C,a^
sample	Viscosity (g.s)			
	JSDR1		JSDR2	JSDR3
XG	−18.8 ± 0.75 ^A,b^		−22.6 ± 0.47 ^B,a^	−26.4 ± 0.55 ^C,a^
G	−14.5 ± 0.47 ^A,a^		−25.1 ± 1.17 ^B,b^	−37.2 ± 1.61 ^C,b^
C	−17.6 ± 0.52 ^A,b^		−21.8 ± 0.86 ^B,a^	−27.7 ± 1.76 ^C,a^
sample		Subsidiary		
		JSDR1	JSDR2	JSDR3
XG		−0.8 ± 0.01 ^A,a^	−0.8 ± 0.03 ^A,a^	−0.7 ± 0.00 ^A,a^
G		−0.7 ± 0.03 ^A,a^	−0.8 ± 0.00 ^B,b^	−0.9 ± 0.01 ^C,c^
C		−0.7 ± 0.02 ^A,a^	−0.8 ± 0.02 ^B,ab^	−0.8 ± 0.02 ^C,b^

* XG: xanthan gum; G: guar bean gum; C: Carrageenan; upper case letters in the same thickener are significantly different (*p* < 0.05); lower case letters in the same JSDR level are significantly different (*p* < 0.05); values are mean ± SD (*n* = 3).

## Data Availability

The raw data supporting the conclusions of this article will be made available by the authors on request.
